# Research on Gut Microbiota Features and Potential Biomarkers in Patients with Pulmonary Tuberculosis

**DOI:** 10.3390/pathogens15080805

**Published:** 2026-07-30

**Authors:** Zi-Jie Chen, Gang Liu, Yuan Wang, Jie-Qing Zhong, Yu-Jie Mo, Dong-Xu Liang, Dan Luo

**Affiliations:** 1 School of Public Health and Management, Guangxi University of Chinese Medicine, 13 Wuhe Avenue, Qingxiu District, Nanning 530200, China; 2 Guangxi Key Laboratory of Translational Medicine for Treating High-Incidence Infectious Diseases with Integrative Medicine, Nanning 530021, China

**Keywords:** pulmonary tuberculosis, gut microbiota, biomarker, 16S rRNA sequencing, feces

## Abstract

(1) Objective: To characterize structural and functional alterations of gut microbiota in patients with newly diagnosed active pulmonary tuberculosis (ATB), screen differential bacterial taxa associated with *Mycobacterium tuberculosis* (MTB) infection, and explore tuberculosis-related microbial metabolic alterations via predictive functional profiling. (2) Methods: Fresh morning fecal samples were collected from 33 treatment-naive patients with newly diagnosed ATB (ATB group) and 30 healthy controls (HC group). 16S rRNA gene high-throughput sequencing was performed to compare intergroup differences in gut microbial α/β diversity, taxonomic composition, and predicted KEGG functional profiles. Receiver operating characteristic (ROC) curve analysis was conducted to evaluate the internal discriminative ability of candidate differential genera in this single small cohort. (3) Results: The ATB group showed significantly lower gut microbial α-diversity than healthy controls (all *P* < 0.05). Principal coordinate analysis (PCoA) based on Bray-Curtis distances combined with permutational multivariate analysis of variance (PERMANOVA) revealed significant overall dissimilarity of gut microbial community structure between the two groups (*R^2^* = 0.31, *p* = 0.005). At the phylum level, the relative abundances of *Firmicutes* and *Bacteroidetes* were higher, while *Proteobacteria* was less abundant in ATB patients relative to HC. At the genus level, *Streptococcus*, *R. gnavus* and *Parabacteroides* were significantly enriched in ATB patients, whereas *Bifidobacterium*, *Pseudomonas*, *Megamonas* and *Faecalibacterium* were depleted. Linear discriminant analysis effect size (LEfSe) analysis uncovered group-specific signature taxa, with pro-inflammatory genera *Streptococcus* and *R. gnavus* markedly enriched in the ATB group. ROC analysis yielded area under the curve (AUC) values of 0.836 for *Streptococcus* and 0.805 for *R. gnavus*. Predictive KEGG functional analysis demonstrated obvious intergroup differences in microbial metabolism, with purine and pyrimidine nucleotide metabolism pathways significantly upregulated in the ATB group. (4) Conclusions: Treatment-naive patients with ATB exhibited reduced gut microbial diversity in this small cohort. The enrichment of *Streptococcus* and *R. gnavus*, as well as the upregulation of purine and pyrimidine metabolic pathways, are all associated with active MTB infection.

## 1. Introduction

Tuberculosis (TB) is a respiratory infectious disease caused by *Mycobacterium tuberculosis* (MTB). It is primarily transmitted via aerosol and is highly contagious, with a propensity for long-term colonization in the lungs, leading to pulmonary tuberculosis (PTB) [1]. According to the World Health Organization (WHO), approximately 10.8 million new TB cases were reported globally in 2023, representing a 4.6% increase since 2020, with about 1.25 million deaths. TB has become the single infectious disease with the highest mortality worldwide, accounting for nearly twice the number of deaths caused by HIV/AIDS [2]. The WHO has proposed the “End TB Strategy”, aiming to reduce incidence by half by 2025 and achieve effective control of TB by 2030. However, progress has fallen far short of these targets. Between 2015 and 2022, the global TB incidence declined cumulatively by only 8.7%, while mortality rates remained persistently high—well below the pace required to meet established goals [2,3].

Pathogen-based detection remains the gold standard for confirming TB; however, the slow growth of MTB results in prolonged detection cycles and low positivity rates. Consequently, clinical diagnosis must integrate symptom assessment, imaging studies, and auxiliary testing [4]. Although several emerging approaches—including immunological assays [5], genomic testing [6], and molecular marker detection [7]—have been developed, limitations in accuracy still contribute to misdiagnosis and missed cases, allowing TB to remain one of the world’s major public health challenges.

Current evidence supporting the “gut–lung axis” suggests that specific gut microbiota and their metabolites can modulate pulmonary immune responses directly or indirectly via systemic circulation [8,9]. Animal studies have demonstrated that MTB-infected mice exhibit marked reductions in the abundance of *Firmicutes* and *Clostridiales* in the gut. Fecal microbiota transplantation or probiotic supplementation can upregulate the expression of immunostimulatory molecules on pulmonary dendritic cells and enhance T-cell activation, thereby restoring anti-TB immunity [10,11]. Clinical investigations further reveal that gut microbiota regulate mucosal-associated invariant T cells (MAIT), which rely heavily on microbial development for their function, and participate in early anti-TB defense. MAIT cells rapidly recognize MTB and collaborate with other innate immune cells to mount an initial protective response. Dysbiosis leads to diminished MAIT cell numbers, weakening the lung’s ability to control primary MTB infection [12,13]. Moreover, during PTB infection, immune cells release large amounts of pro-inflammatory cytokines, triggering systemic inflammation and increasing intestinal vascular permeability, which alters the gut microenvironment [14]. In addition, microbial metabolites such as indole-3-propionic acid have been shown to inhibit key MTB synthetic enzymes, exerting antibacterial effects [15].

Consistent with the accumulating body of gut–lung axis evidence linking intestinal microecology to MTB infection, the present cross-sectional study utilized 16S rRNA high-throughput sequencing on fecal specimens collected from patients with ATB and HC participants. We systematically compared intergroup disparities in gut microbial diversity, taxonomic relative abundance, and predicted functional metabolic pathways, and screened differential microbial taxa with statistical capacity to separate the two cohorts. The observational data generated herein may deliver preliminary microecological evidence to inform non-invasive auxiliary discrimination and gut microbiota-targeted interventional research for TB.

## 2. Subjects and Methods

### 2.1. Study Population

A 1:1 case–control design was used. From January to December 2024, 33 patients with active pulmonary tuberculosis (ATB group) were prospectively recruited from the People’s Hospital of Pingnan County, Guigang City, Guangxi Zhuang Autonomous Region, China. For each case, all healthy controls (HC group) were recruited from the same county-level hospital physical examination center as ATB patients, covering identical residential areas. The two groups were matched based on all available baseline variables to improve intergroup comparability. Fresh fecal samples (100 mg) from the two groups were obtained in the morning to minimize the impact of confounding factors, then frozen instantly in liquid nitrogen and stored at −80 °C. Treatments or preventive therapy were administrated after fecal samples were collected. Three HC participants were excluded due to failure to meet sequencing quality requirements, resulting in 30 individuals in the final analysis. Inclusion criteria for the ATB group: (1) meets the Diagnostic Criteria for Pulmonary Tuberculosis (WS 288-2017) [16]; (2) newly diagnosed, treatment-naïve pulmonary TB; (3) age ≥ 18 years. Exclusion criteria for the ATB group: (1) immunodeficiency disorders; (2) any use of antibiotics or immunosuppressants within 1 month before enrollment; (3) pregnancy. Inclusion criteria for the HC group: (1) no cough, expectoration, or other symptoms suggestive of TB, and no history of TB; (2) no serious underlying cardiovascular, hepatic, or renal diseases; (3) age ≥ 18 years. Exclusion criteria for the HC group: (1) any use of antibiotics or immunosuppressants within 1 month before enrollment; (2) history of malignancy or other severe illness; (3) pregnancy; (4) extrapulmonary TB.

### 2.2. 16S rRNA Gene Sequencing

Genomic DNA was extracted from fecal samples using the cetyltrimethylammonium bromide (CTAB) method and assessed by 1% agarose gel electrophoresis. DNA was diluted to 1 ng/μL, and the V3 – V4 region of the 16S rRNA gene was amplified using barcoded primers 341F/806R. PCR reactions included Phusion High-Fidelity Master Mix, 0.2 μM of each primer, and 10 ng template DNA, with thermal cycling consisting of initial denaturation at 98 °C for 1 min, followed by 30 cycles of 98 °C/10 s, 50 °C/30 s, 72 °C/30 s, and a final extension at 72 °C for 5 min.

Amplicons were visualized on 2% agarose gels, pooled, and purified using a QIAquick Gel Extraction Kit (Qiagen, Hilden, Germany). Sequencing libraries were prepared with the NEBNext Ultra II DNA Library Prep Kit (New England Biolabs, Ipswich, MA, USA), and quality was verified using a Qubit 2.0 Fluorometer (Thermo Fisher Scientific, Waltham, MA, USA) and Agilent Bioanalyzer 2100 (Agilent Technologies, Santa Clara, CA, USA). Libraries were sequenced on an Illumina NovaSeq platform (Illumina, San Diego, CA, USA) to generate 250 bp paired-end reads.

Raw reads were merged using FLASH (v1.2.11) and trimmed with fastp (v0.20.0). Chimeric sequences were identified and removed using VSEARCH (v2.15.0) against the SILVA database to obtain high-quality amplicon sequence variants (ASVs), with low-abundance ASVs (<5 counts) filtered out. Data were rarefied to the minimum sequencing depth across samples. Taxonomic annotation was performed using the SILVA database implemented in QIIME 2 (v2020.6).

### 2.3. Bioinformatics Analyses of the Microbiome

α-diversity indices were calculated using QIIME 2 (v 2020.6). Intergroup comparisons were performed using the Wilcoxon rank-sum test in SPSS 23. β-diversity was evaluated through principal coordinate analysis (PCoA) based on the Bray–Curtis dissimilarity matrix to examine community structure variation, and permutational multivariate analysis of variance (PERMANOVA) was applied to test for significant taxonomic differences between groups.For differential genus identification, only taxa annotated at the genus level with an absolute abundance > 0 in at least 70% of the samples were considered. Significant intergroup differences in genus-level abundance were determined using the Wilcoxon rank-sum test in R (packages: rstatix, dplyr) with *p* values corrected for multiple testing using the Benjamini–Hochberg method (false discovery rate [FDR] < 0.05). Linear discriminant analysis effect size (LEfSe) was adopted to screen differential microbial biomarkers between groups, with the Linear Discriminant Analysis (LDA) score threshold set to 4 to identify taxa with strong discriminatory power. Receiver operating characteristic (ROC) curves were subsequently utilized to evaluate the discriminatory efficiency of differential genera. Based on the ASV abundance matrix and taxonomic classification against the SILVA database, the Tax4Fun pipeline was employed to predict functional profiles of the gut microbiota, with functional annotations referenced to the KEGG database.All statistical tests were two-tailed, and the significance level was set at α = 0.05.

### 2.4. Statistical Analyses

Statistical analyses were conducted with IBM SPSS Statistics 23 (IBM Corp., Armonk, NY, USA). Normally distributed continuous variables were presented as mean ± standard deviation (*SD*) and compared using the independent samples t-test; non-normally distributed continuous variables were reported as median (interquartile range, *IQR*) and analyzed using the Wilcoxon rank-sum test. Categorical variables were summarized as percentages, and intergroup comparisons were carried out with the chi-squared test. A two-tailed *p* < 0.05 was defined as the threshold for statistical significance.

## 3. Results

### 3.1. Baseline Characteristics of the Study Participants

As shown in Table 1, there were no statistically significant differences between the ATB and HC groups in key baseline characteristics, including age, sex, body mass index (BMI), ethnicity, occupation type, lifestyle habits, and the presence of relevant underlying medical conditions (all *p* > 0.05).

### 3.2. Gut Microbiota Sequencing Data and ASV Clustering

**Figure 1:**
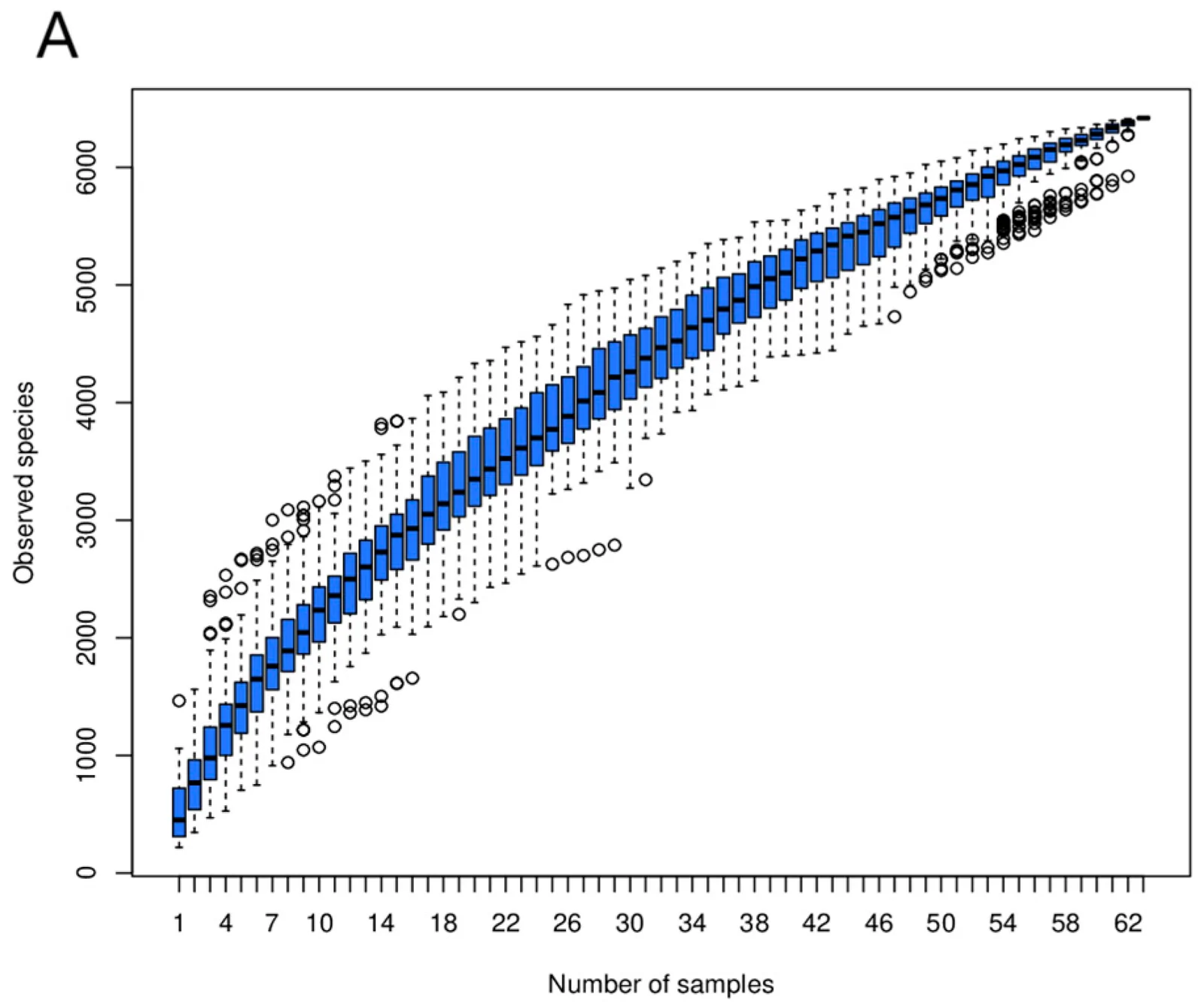

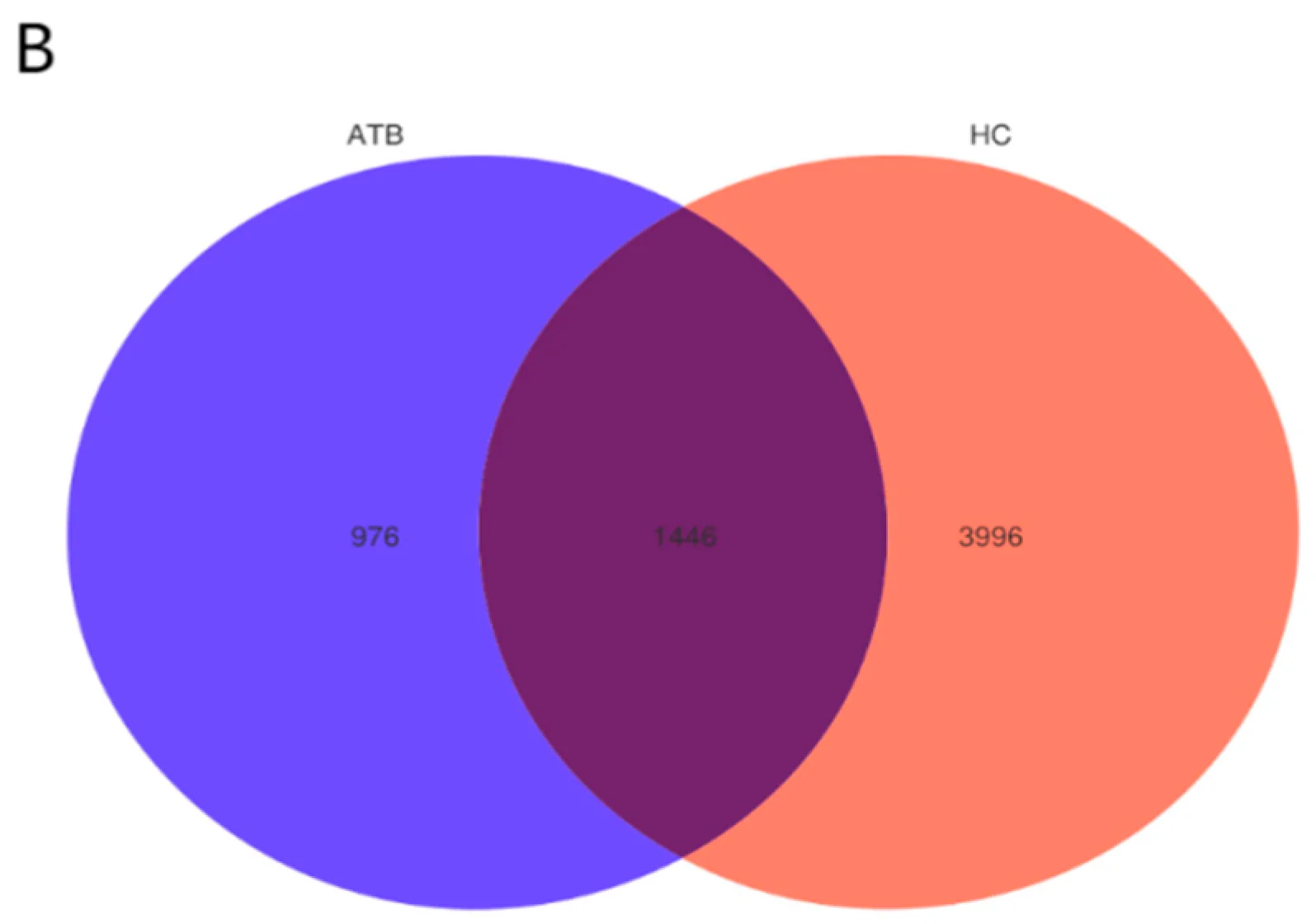
(**A**) Species accumulation box plot showing the trend of newly detected ASVs with increasing sample size. The curve plateaued, indicating that the current sample size adequately captured the majority of microbial diversity. (**B**) Venn diagram illustrating the overlap and uniqueness of ASVs between the ATB and HC groups.

High-throughput sequencing of the V3 – V4 region of the 16S rRNA gene was performed on fecal samples from 63 study participants, generating a total of 5,133,651 raw sequences. After sequence assembly, quality control, and filtering, 4,674,194 high-quality sequences were retained, with an effective rate of 91% (an average of 74,194 sequences per sample). Following denoising and deduplication, a total of 6418 ASVs were identified. As the sample size increased, the rate of detecting new species gradually slowed, and the accumulation curve reached a plateau, indicating that the current sample size was sufficient to comprehensively represent the overall species composition (Figure 1A). Further analysis revealed that 1446 ASVs (22.5%) were shared between the ATB and HC groups. The ATB group had 976 unique ASVs (15.2%), while the HC group had 3996 unique ASVs (62.3%) (Figure 1B).

### 3.3. Analysis of Gut Microbiota Diversity

α-diversity analyses illustrated that ATB patients exhibited markedly lower Chao1, Observed OTUs, Shannon and Simpson indices relative to HC, with all *p* values less than 0.05 (Table 2). 

**Figure 2:**
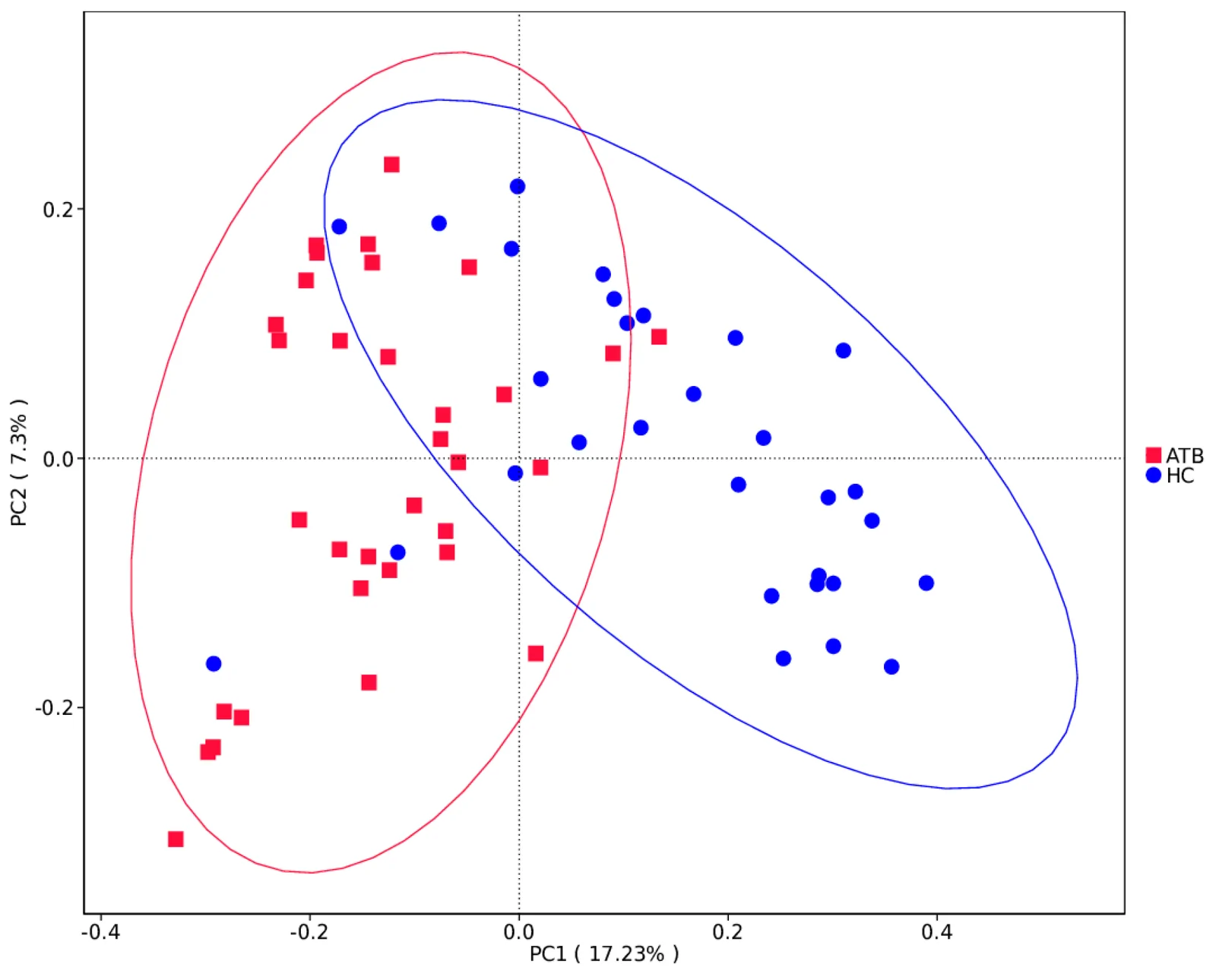
PCoA of Gut Microbiota β-Diversity Based on Bray–Curtis Dissimilarity. Samples from the ATB group (active tuberculosis, red squares) and HC group (healthy controls, blue dots) are plotted along the first two principal coordinates (PCoA1 and PCoA2). PCoA: Principal Coordinates Analysis—a dimensionality reduction method used to visualize dissimilarity matrices (here, Bray–Curtis) in 2D space. Bray–Curtis dissimilarity: A measure of community dissimilarity based on species abundance, ranging from 0 (identical communities) to 1 (completely different). PERMANOVA: Permutational Multivariate Analysis of Variance, a permutation-based nonparametric approach to test intergroup divergence in multivariate community composition.

For β-diversity profiling, PCoA revealed that the top two principal axes (PCoA1 and PCoA2) accounted for 17.23% and 7.30% of the total variance in gut microbial community configuration, respectively. While partial overlap was observed between the microbial profiles of the two cohorts, their global spatial distributions displayed evident segregation, which implied profound intergroup dissimilarities in overall microbiota architecture (Figure 2). Subsequent PERMANOVA further validated the statistically significant compositional divergence between ATB and HC groups (*R^2^* = 0.31, *p* = 0.005).

### 3.4. Gut Microbiota Composition Characteristics

**Figure 3:**
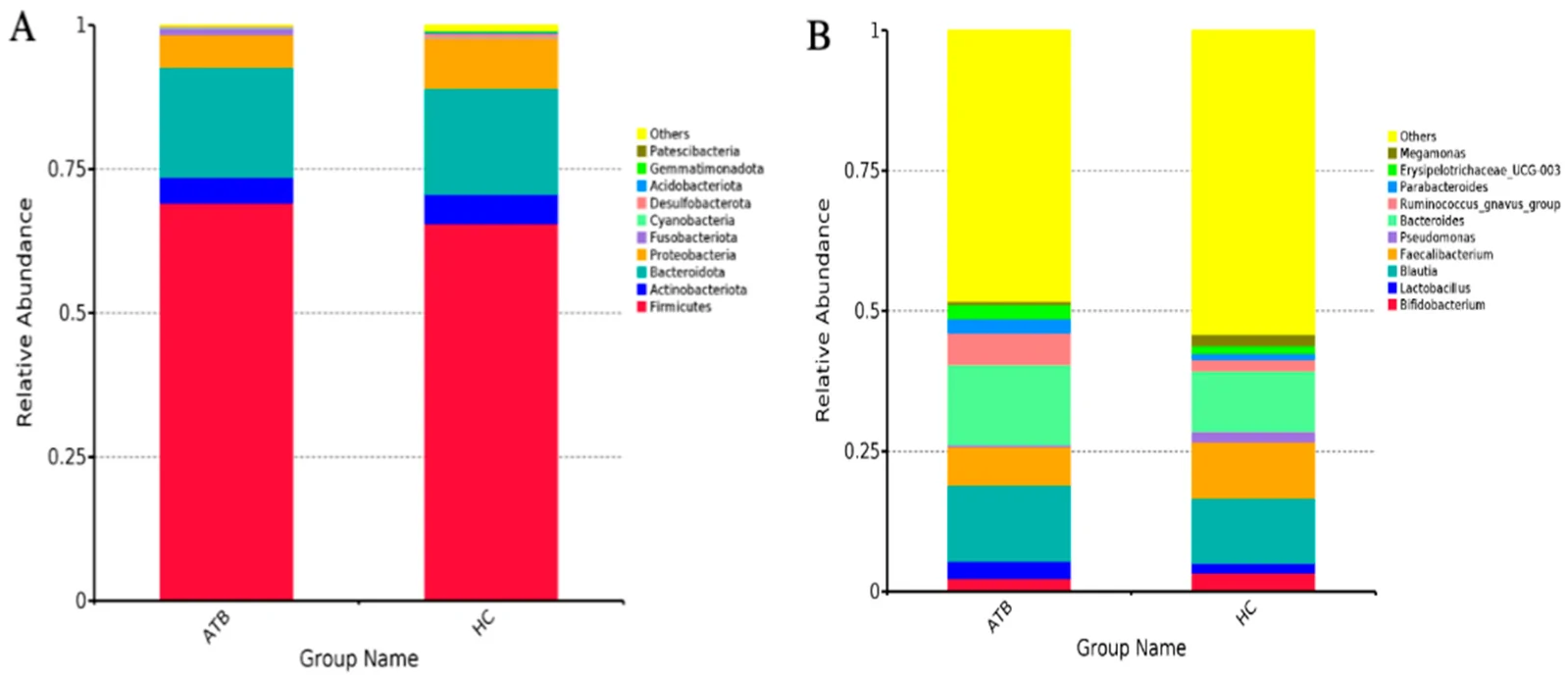
Comparison of gut microbiota relative abundance between the two groups. (**A**) Phylum-level composition: stacked bar plot showing the relative abundance of major bacterial phyla in the ATB and HC groups. (**B**) Genus-level composition: stacked bar plot illustrating the relative abundance of key bacterial genera. Taxonomic groups are color-coded according to the legend; “Others” represent low-abundance taxa combined into a single category.

At the phylum level, the dominant phyla in both groups were *Firmicutes*, *Bacteroidota*, and *Proteobacteria*. Compared with the HC group, the ATB group exhibited significantly higher relative abundances of *Firmicutes* and *Bacteroidota*, but a lower relative abundance of *Proteobacteria* (Figure 3A).

At the genus level, the intergroup abundance differences of 8 genera remained statistically significant (all *p* < 0.05), including enriched *Streptococcus*, *Ruminococcus_gnavus_group* (*R. gnavus*), *Parabacteroides* and depleted *Bifidobacterium*, *Faecalibacterium*, *Pseudomonas*, *Megasphaera*, *Holdemania* in ATB patients. Non-significant genera such as *Blautia*, *Lactobacillus*, *Megamonas* and *Erysipelotrichaceae_UCG003* showed no consistent intergroup disparity. Details are provided in Figure 3B and Table 3.

### 3.5. Differential Species Analysis of Gut Microbiota

**Figure 4:**
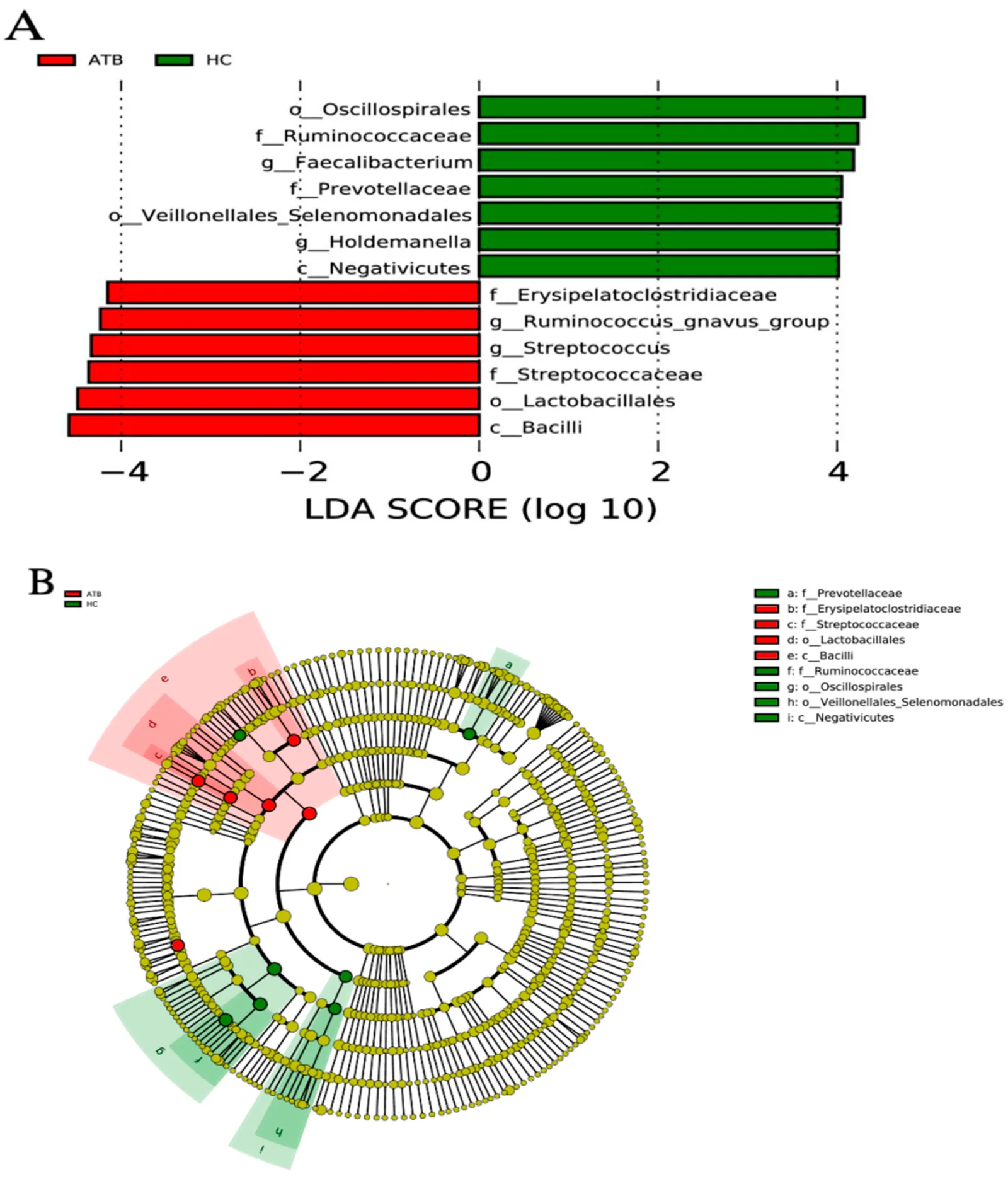
LEfSe Analysis of Gut Microbiota Between ATB and HC Groups. (**A**) LDA Score Plot: The horizontal axis represents the LDA score (log10 scale), and the vertical axis lists the significantly different bacterial taxa (at the genus level) identified by LEfSe. Taxa with LDA scores > 4 are shown, with red indicating taxa enriched in the ATB group and green indicating taxa enriched in the HC group. (**B**) Evolutionary Branch Plot: The circular cladogram illustrates the phylogenetic relationships of the gut microbiota. Different colors (red for ATB—enriched taxa, green for HC—enriched taxa) mark the taxonomic groups with significant abundance differences, visually reflecting the evolutionary distribution of these differential taxa within the microbial community.

Using LEfSe analysis at the genus level, the following significantly different taxa were identified: in the ATB group, *Streptococcus* (LDA = 4.75) and *R. gnavus *(LDA = 4.74); in the HC group, *Faecalibacterium* (LDA = 4.99) and *Holdemanella* (LDA = 4.35), as shown in Figure 4.

### 3.6. Exploratory Group Differentiation Analysis of Key Differential Taxa

**Figure 5:**
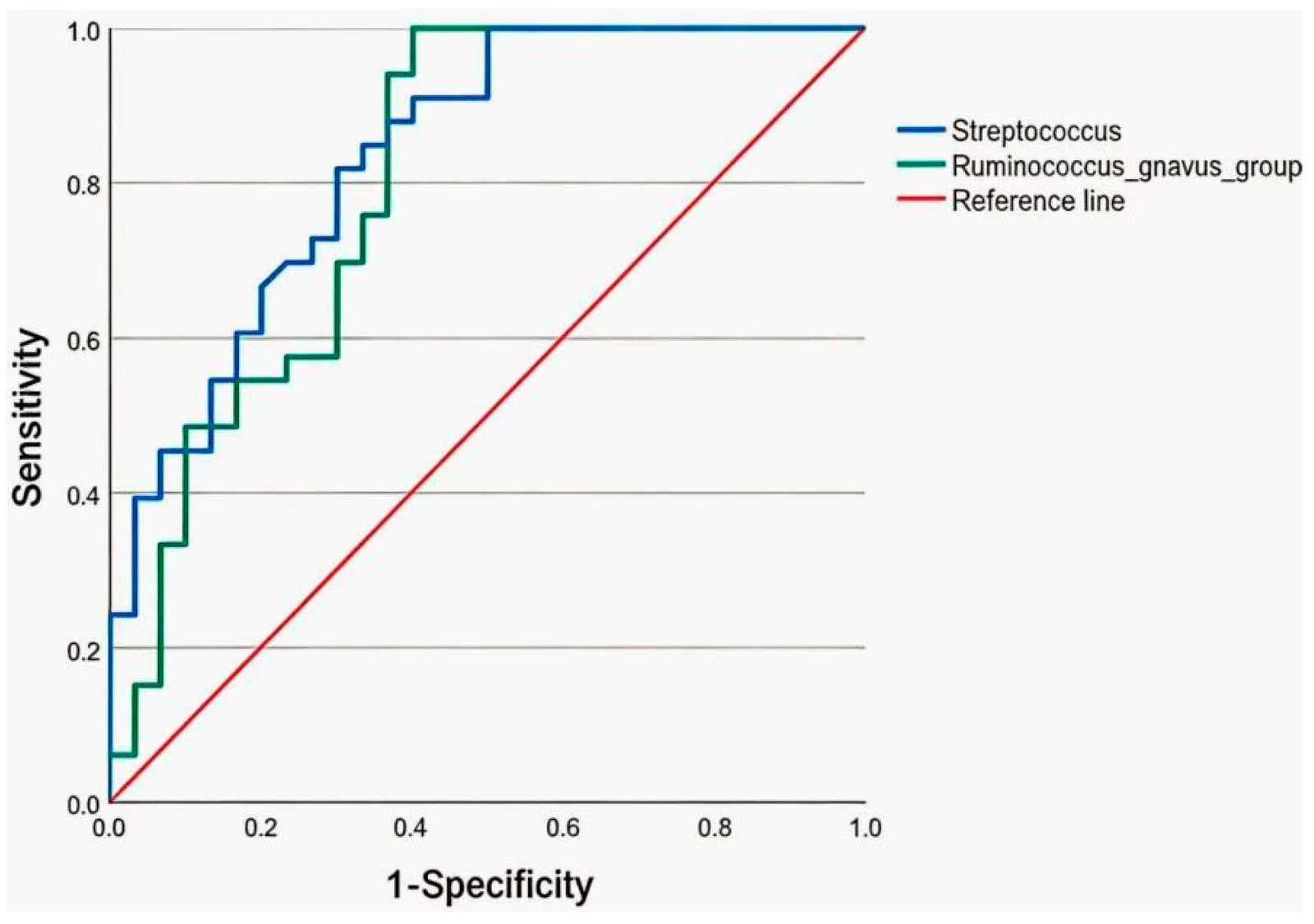
ROC curves illustrate the intergroup discrimination capacity of two signature bacterial taxa identified by LEfSe in separating ATB patients from HC. Both curves lie above the reference line (red diagonal), indicating good discriminatory ability. The AUC quantifies the group separation performance, with values closer to 1.0 reflecting stronger capacity to distinguish the two populations.

ROC curve analysis was conducted to preliminarily assess the intergroup distinguishing capacity of taxonomic biomarkers screened via LEfSe between ATB patients and HC. The calculated area under the curve (AUC) values of *Streptococcus* (AUC = 0.836) and *R. gnavus* (AUC = 0.805) suggested moderate capacity to separate ATB patients from HC (Figure 5).

### 3.7. Gut Microbiota Functional Analysis

**Figure 6:**
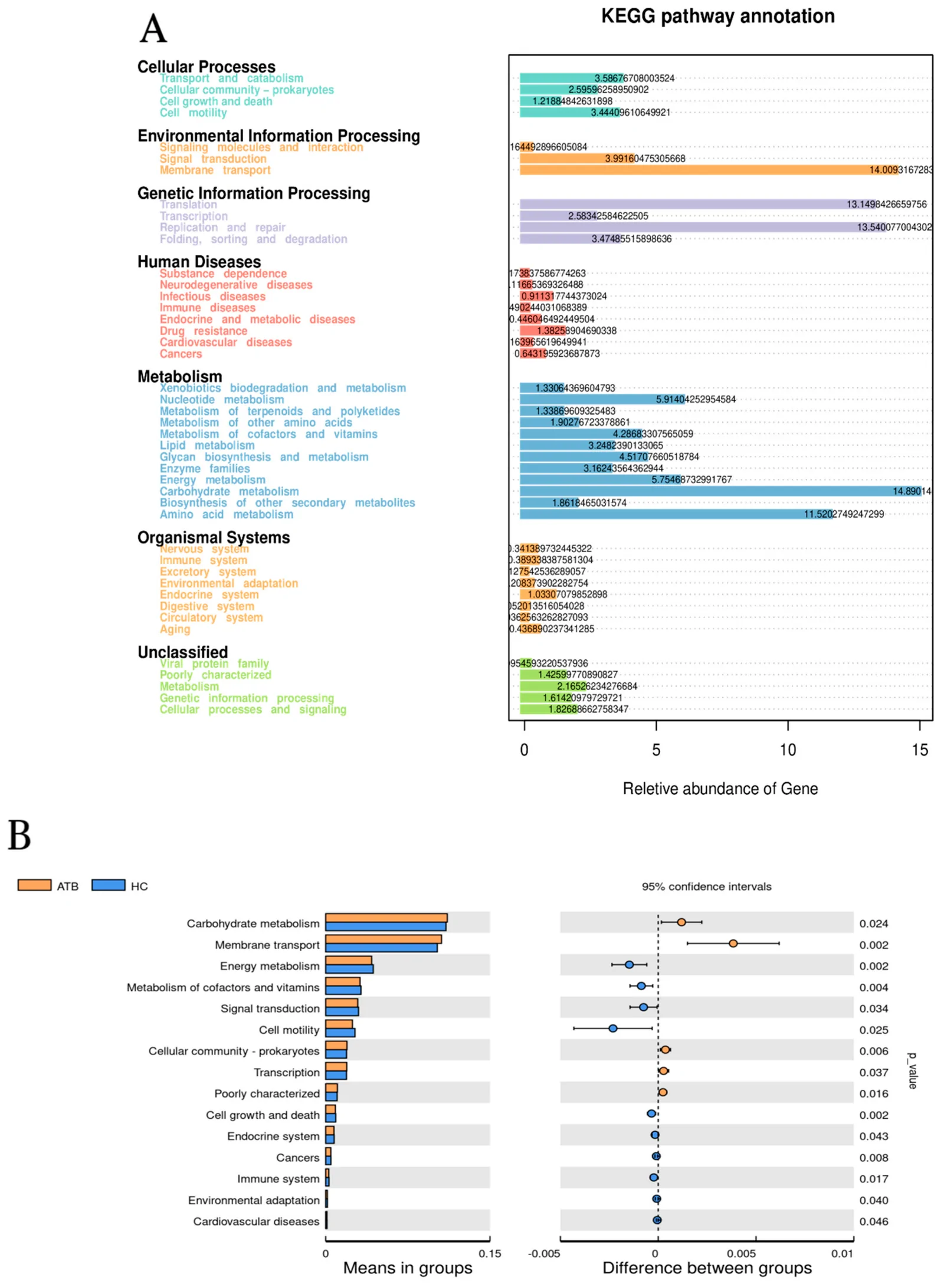

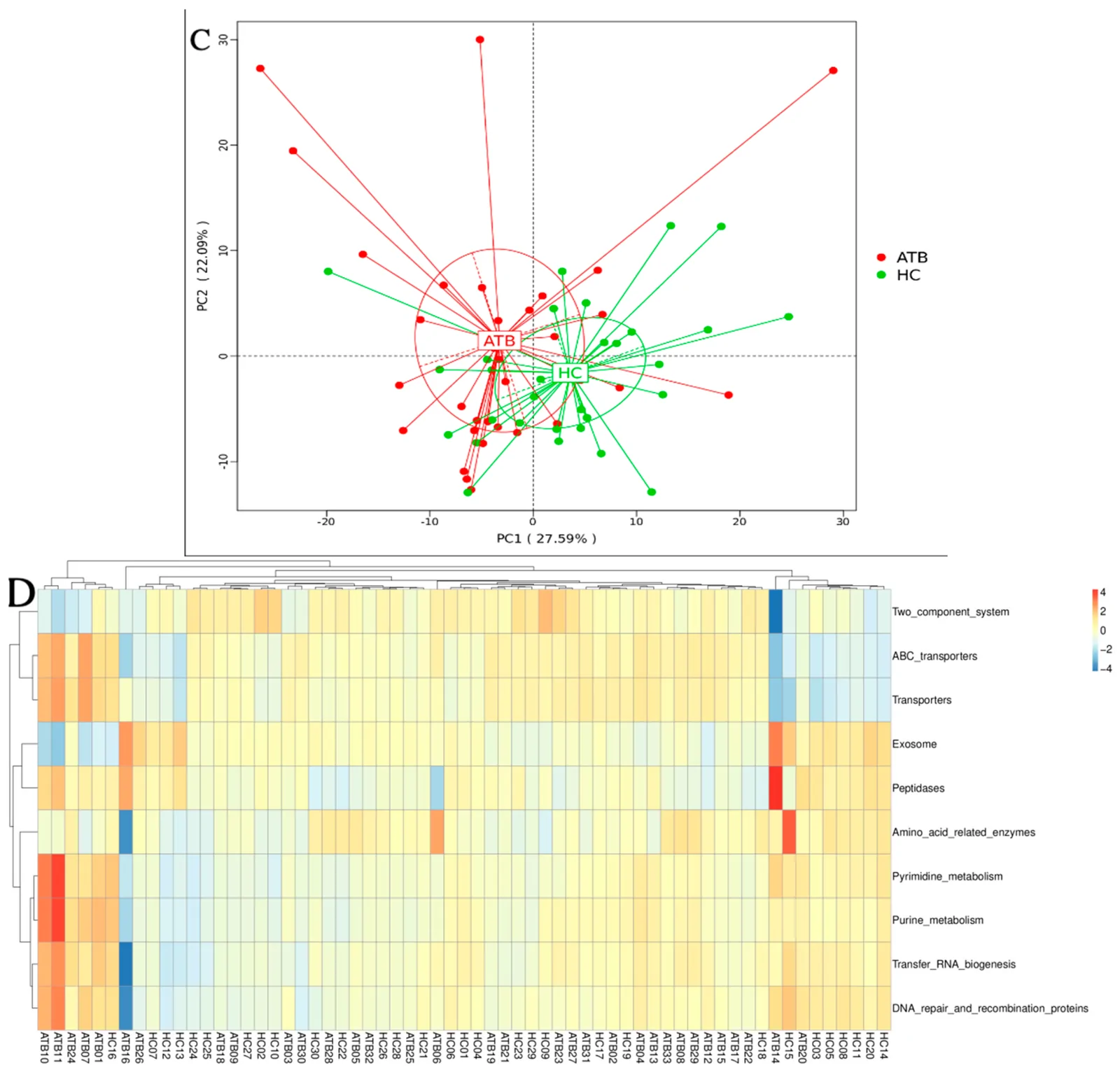
KEGG Functional Analysis of Gut Microbiota. (**A**) First-level KEGG Functional Pathway Annotation. (**B**) Second-level Differential Functional Metabolic Pathways. (**C**) PCoA Analysis of Differential Functional Pathways. (**D**) Heatmap of Top 10 Differentially Abundant Third-level Metabolic Pathways.

Based on Tax4Fun functional prediction and KEGG database annotation, six core metabolic pathways were identified at the first hierarchical level, including: metabolism, genetic information processing, environmental information processing, human diseases, cellular processes, and organismal systems. Among these, carbohydrate metabolism and membrane transport showed the highest relative abundances (Figure 6A). At the second hierarchical level, a total of 15 pathways exhibited significant differences between groups (all *p* < 0.05). Of these, five pathways had significantly higher abundances in the ATB group compared with the HC group (details in Figure 6B). Furthermore, PCoA revealed clear functional differences in gut microbiota between the two groups (Figure 6C). Cluster analysis was performed on the top 10 most differentially abundant pathways at the third hierarchical level. Notably, Purine metabolism and Pyrimidine metabolism were significantly upregulated in pulmonary tuberculosis patients (Figure 6D).

## 4. Discussion

TB remains a severe infectious disease that imposes a heavy global public health burden, especially within low- and middle-income developing countries.A major obstacle to effective TB control lies in the scarcity of simple, robust diagnostic tools. Against this backdrop, the discovery of non-invasive gut microbial biomarkers to distinguish ATB patients from HC carries profound research value. Mounting evidence has revealed striking compositional divergences in gut microbiota between ATB patients and healthy individuals. Profiling such gut microbial discrepancies between the two cohorts is therefore indispensable to decipher how gut microbes mediate the onset and advancement of tuberculosis.

At the phylum level, the gut microbial communities of both ATB patients and HC were predominated by *Firmicutes*, *Bacteroidetes* and *Proteobacteria*. Relative to healthy participants, ATB patients exhibited elevated proportional abundances of *Firmicutes* and *Bacteroidetes*, alongside a reduction in *Proteobacteria*. Such taxonomic shifts may correlate with MTB invasion, disrupted intestinal homeostasis or impaired host physical status, yet causal links cannot be established based solely on this cross-sectional observation. Consistent with previously published evidence [17,18], ATB patients displayed markedly lower gut microbial α-diversity, and PCoA coupled with PERMANOVA confirmed significant overall compositional dissimilarity between the two groups, a pattern likely associated with MTB-induced remodeling of the intestinal microbial ecosystem.

Short-chain fatty acids (SCFAs) modulate host immune responses through multiple biological mechanisms, exerting antibacterial effects, maintaining intestinal epithelial barrier integrity and supporting host energy metabolism [19]. Among the genera with statistically significant intergroup disparities, *Bifidobacterium*, *Faecalibacterium*, *Megamonas* and *Parabacteroides* are well-documented SCFA-producing taxa [20,21,22]. In our dataset, all four genera except *Parabacteroides* were depleted in the ATB group. Existing mechanistic studies have demonstrated that *Bifidobacterium* reinforces host anti-MTB defenses by boosting antibody secretion, IFN-γ production and NK cell activation, while alleviating pulmonary inflammatory damage via facilitating Treg cell differentiation [23,24]. The depletion of SCFA-producing taxa, particularly *Bifidobacterium*, in ATB patients may disrupt intestinal microecology, which correlates with aggravated systemic inflammation and attenuated antimycobacterial immune capacity, matching previous observational findings linking reduced SCFA producers to MTB infection [25]. Notably, *Parabacteroides*, another SCFA-producing genus, were enriched in our ATB group. Higher abundance of this taxon has also been reported in patients with ankylosing spondylitis and gestational diabetes mellitus [26,27], implying that the overgrowth of *Parabacteroides* may correlate with chronic inflammatory states rather than being MTB-specific. Additionally, certain studies have reported context-dependent adverse effects of SCFAs under specific comorbid conditions: among patients co-infected with tuberculosis and diabetes, butyrate elevates IL-10 levels to suppress pro-inflammatory responses, which may impair host clearance of MTB and increase infection susceptibility [28]. Collectively, the functional roles of SCFA-generating bacteria in ATB and tuberculosis-related comorbidities require more targeted experimental validation.

Two genera with consistent intergroup separation signals were identified in the ATB group: *Streptococcus* and *R. gnavus*. *Streptococcus* represents a common opportunistic human pathogen capable of triggering pathological conditions ranging from mild upper respiratory tract infections to severe suppurative and hypersensitivity disorders [29]. Preclinical animal models have validated that streptococcal infection triggers severe pulmonary injury, disrupts alveolar epithelial barrier tight junctions and modulates macrophage autophagic activity in mice [30]. Consistent with prior cross-sectional research, untreated ATB patients show intestinal enrichment of *Streptococcus*. Longitudinal follow-up data further revealed a negative correlation between post-treatment *Streptococcus* abundance and serum albumin concentrations among tuberculosis patients [31]. Meanwhile, long-term loss of beneficial gut bacteria and continuous expansion of pro-inflammatory microbes break gut microecological balance and interfere with the host’s uptake of energy and micronutrients [32], an association that may reflect bidirectional interactions between intestinal streptococcal expansion and host nutritional–immune homeostasis during infection. *R. gnavus* participates in the regulation of multiple systemic physiological axes via diverse molecular mediators [33]. Its cell-surface polysaccharides can activate dendritic cells through the TLR4 signaling cascade to drive the secretion of pro-inflammatory cytokines such as TNF-α, amplifying intestinal inflammatory responses [34]. Longitudinal clinical data have recorded declining intestinal *R. gnavus* levels in ATB patients following standard anti-tuberculosis chemotherapy [25]. Independent metabolomic analyses have reported negative correlations between *R. gnavus* abundance and intestinal inosine, xanthosine, xanthine and hypoxanthine, alongside positive associations with the inosine degradation sub-pathway (adenosine nucleotide degradation II), supporting the involvement of *R. gnavus* in intestinal purine metabolism [35]. Cumulatively, taxonomic remodeling of specific gut microbes may modulate nutrient absorption and host metabolic homeostasis, which correlates with the progression and therapeutic outcomes of MTB infection.

KEGG functional pathway prediction identified robust differences in gut microbial metabolic profiles between ATB patients and HC. Pathways involved in nucleotide metabolism, namely purine metabolism and pyrimidine metabolism, which participate in DNA/RNA biosynthesis, energy transduction and intracellular signal transduction, were significantly enriched in the ATB group. Multiple prior studies have confirmed the immunomodulatory functions of purine and pyrimidine metabolites. The enzyme ISOC1 within Th1 lymphocytes interferes with intracellular pyrimidine metabolism to suppress the secretion of IFN-γ and IL-17; individuals with impaired IFN-γ synthesis demonstrate increased susceptibility to mycobacterial diseases [36,37]. Hypoxanthine-guanine phosphoribosyltransferase acts as a core enzyme in the purine salvage pathway, and its inhibitors have been proposed as candidate anti-mycobacterial agents by disrupting MTB purine utilization [38]. The observed upregulation of purine and pyrimidine metabolic pathways therefore correlates with the status of active MTB infection.

However, the present study also has some limitations.First, as a small-sample cross-sectional case–control study, this work only identifies correlational associations between gut microbiota and active tuberculosis, yet it cannot clarify the causal relationship between intestinal dysbiosis and MTB infection. Large-scale prospective cohort investigations are required to differentiate whether baseline gut flora imbalance elevates TB susceptibility or arises as a secondary consequence after MTB infection. Second, all differential taxa and metabolic pathways were computationally predicted from 16S amplicon data, lacking multi-omics and in vitro/in vivo functional validation. Follow-up experiments will be performed to validate these microbial signatures. Third, we strictly excluded subjects exposed to antibiotics, anti-TB drugs and probiotics via enrollment criteria, yet long-term residual effects of past medications and minor interference from other drugs could not be fully eliminated, potentially introducing analytical bias. Fourth, no standardized unified diet was adopted for participants. Although all subjects resided in the same county with consistent dietary habits, diet broadly reshapes gut microbiota, representing an additional limitation of this study.

## 5. Conclusions

In summary, this cross-sectional 16S rRNA sequencing study systematically compared gut microbial profiles between untreated ATB patients and healthy volunteers. We observed marked intestinal dysbiosis in ATB group, characterized by reduced microbial α-diversity and altered overall community composition, and screened two specific differential genera closely associated with MTB infection status.Functional prediction also uncovered infection-associated shifts in microbial nucleotide metabolic pathways. These findings provide preliminary observational evidence to support the development of non-invasive intestinal microbial auxiliary discrimination tools and microecological intervention strategies for TB, with clear limitations that must be acknowledged.

## Figures and Tables

**Table 1:** Baseline Characteristics of Study Participants.

Characteristics		Study Grouping		Statistical Value	*p* Value
		ATB Group (*n* = 33)	HC Group (*n* = 30)		
Age (years)		48.3 ± 12.9	47.4 ± 13.2	0.277 ^a^	0.783
BMI (kg/m^2^)		22.1 ± 3.2	21.8 ± 2.9	0.389 ^a^	0.699
Gender	Male	15 (45.5)	14 (46.7)	0.009 ^b^	0.923
	Female	18 (54.6)	16 (53.3)		
Ethnicity	Han	28 (84.8)	26 (86.7)	0.024 ^b^	0.877
	Other	5 (15.2)	4 (13.3)		
Occupation	Farmer	18 (54.5)	15 (50.0)	0.130 ^b^	0.718
	Other	15 (45.5)	15 (50.0)		
Smoking	Yes	9 (27.3)	6 (20.0)	0.458 ^b^	0.498
	No	24 (72.7)	24 (80.0)		
Alcohol Consumption	Yes	11 (33.3)	8 (26.7)	0.332 ^b^	0.565
	No	22 (66.7)	22 (73.3)		
Comorbid Diabetes Mellitus	Yes	4 (12.1)	2 (6.7)	-	0.681
	No	29 (87.9)	28 (93.3)		
Comorbid HIV Infection	Yes	0 (0.0)	0 (0.0)	-	-
	No	33 (100.0)	30 (100.0)		

Notes: (1) Quantitative data are presented as mean ± SD. Qualitative data are expressed as counts and percentages (%). (2) BMI: body mass index. (3) For the item “Comorbid Diabetes Mellitus,” because two theoretical frequencies were< 5, Fisher’s exact test was used to calculate *p*-values. (4) For the item “Comorbid HIV Infection,” no positive cases were observed in either group, so no statistical test was performed. (5) In the table, “^a^” denotes t-value, and “^b^” denotes chi-square value.

**Table 2:** Comparison of α-diversity indices.

Group	Chao1	Observed_otus	Shannon	Simpson
ATB Group	322.01 (287.55, 356.46)	318.52 (284.66, 352.37)	5.42 ± 0.12	0.93 (0.91, 0.94)
HC Group	649.35 (554.93, 743.76)	639.40 (545.01, 733.79)	6.31 ± 0.14	0.96 (0.95, 0.97)
*Z* value	5.725	5.725	4.129	3.262
*p* value	<0.001	<0.001	<0.001	0.001
FDR-*P* value	<0.001	<0.001	<0.001	0.002

Notes: Chao1: An index estimating species richness (total number of species) by accounting for rare species. Observed_OTUs: The number of operational taxonomic units (OTUs) or ASVs directly observed in the sample, reflecting the total number of distinct microbial taxa at a given similarity threshold (e.g., 97% for OTUs, 100% for ASVs). Shannon: A diversity index integrating both species richness and evenness (relative abundance distribution). Simpson: A diversity index emphasizing dominance (lower values = higher diversity). Data in the table are expressed as mean ± SD or median (*IQR*). The statistical “Z-value” corresponds to the Z-statistic of the Mann–Whitney U test (due to the relatively large sample size, the U-test approximates a normal distribution, so the Z-value is reported instead).

**Table 3:** Comparison of genus-level relative abundance of gut microbiota between the two groups.

Genus	Relative Abundance (%)		Z Value	* p * Value	FDR- * P * Value
	ATB Group	HC Group			
*Bifidobacterium*	2.38	3.35	3.138	0.002	0.004
*Blautia*	13.56	11.62	−0.482	0.630	0.682
*Lactobacillus*	3.07	1.73	−1.356	0.175	0.214
*Faecalibacterium*	6.78	9.93	2.863	0.004	0.007
*Pseudomonas*	0.36	1.86	2.471	0.013	0.021
*Megamonas*	14.34	10.90	−0.812	0.417	0.475
*Erysipelotrichaceae_UCG003*	2.56	1.32	0.227	0.820	0.851
*R. gnavus*	5.58	1.95	−4.156	<0.001	<0.001
*Parabacteroides*	2.57	1.18	−2.629	0.009	0.016
*Megasphaera*	0.50	1.97	3.757	<0.001	<0.001
*Streptococcus*	2.64	0.80	−4.569	<0.001	<0.001
*Holdemania*	0.07	0.76	5.586	<0.001	<0.001

Note: The statistical “Z-value” corresponds to the Z-statistic of the Mann–Whitney U test (due to the relatively large sample size, the U-test approximates a normal distribution, so the Z-value is reported instead).

## Data Availability

The original dataset of this study can be obtained by reasonable request from the corresponding author: Dan Luo, Email: luod2017@gxtcmu.edu.cn.

## Abbreviations

The following abbreviations are used in this manuscript:

MTB	Mycobacterium tuberculosis
TB	Tuberculosis
PERMANOVA	Permutational multivariate analysis of variance
PCoA	Principal coordinate analysis based on UniFrac distance
LEfSe	Linear discriminant analysis effect size
MAIT	Mucosal-associated invariant T cell
ASV	Amplicon sequence variant
TLR4	Toll-like receptor 4
ROC curve	Receiver operating characteristic curve
AUC	Area under the curve
95% CI,	95% confidence interval
LDA	Linear Discriminant Analysis
SCFAs	Short-Chain Fatty Acids
KEGG	Kyoto Encyclopedia of Genes and Genomes
Th1	T helper cell 1
ISOC1	Isoleucyl-tRNA Synthetase Cytoplasmic 1
IL-17	Interleukin 17
TNF-α	Tumor Necrosis Factor α
IFN-γ	Interferon γ
